# It May Be Possible to Prevent both Early and Late Relapses in Breast Cancer; Perhaps This Is an Opportunity for Sensors or Biosensors to Help

**DOI:** 10.3390/s20247261

**Published:** 2020-12-18

**Authors:** Michael Retsky

**Affiliations:** Harvard TH Chan School of Public Health, Boston, MA 02115, USA; Michael.retsky@gmail.com

Data presented in 1993 showed an unexpected bimodal relapse pattern in surgically treated breast cancer. There was an early wave of relapses in the first 3 years after primary surgery, then a minimum at 4 years followed by a late wave extending from 5 to over 10 years. This was unexplainable with the current cancer paradigm that has long guided cancer therapy. After much analysis and input from a number of scientific and medical specialists, my colleagues and I eventually came to the conclusion that the surgery to remove the primary tumor produces systemic inflammation (SI) for a week after surgery. This SI causes exits of cancer from dormant states resulting in relapses in the first 3 years post-surgery. It was later found in a retrospective study that the common perioperative analgesic non-steroidal anti-inflammatory drug (NSAID) ketorolac could prevent the early relapses. This has been confirmed in a second retrospective study and supported in animal models but not yet in a prospective study. Based on insight from Dillekas et al., 2016, there may be an equivalent method of preventing late relapses. Measuring SI in subjects without needing an invasive blood draw would be very helpful. Presenting this information in a *Sensors* journal on its 20th anniversary is done to stimulate new ideas on less invasive ways of measuring SI.

Breast cancer is the most common female cancer and appears in every country. There are approximately 2 million new breast cancers diagnosed yearly in the world. After diagnosis of early-stage breast cancer, the primary tumor is removed and if the prognosis is less than very favorable, the patient is given adjuvant therapy for approximately 6 months with intent to prevent metastatic relapse—the common pathway to death from the disease. This is only partially successful in that this year 276,000 new invasive breast cancers were diagnosed, and 42,000 patients will die of the disease in the US despite widespread use of aggressive efforts to find cancer early and treat intensely as soon as possible.

In a recent paper, my colleagues and I proposed a method to prevent early relapses in breast cancer [1]. (For a well-done graphic perspective, see the Moss report [2].) This method to prevent early relapses has been tested in two retrospective studies and is supported by experiments in two animal model studies [3,4,5,6]. There are also clinical reports that this method seems to work in colon cancer and ovarian cancer [7,8,9]. This still needs to be confirmed in one or more prospective randomized controlled clinical trial and we are working to have that underway. Meanwhile, it is reasonable to ask that since we have apparently learned how to prevent early relapses, why cannot an equivalent method be found to prevent late relapses?

Recent Development: It may be possible to prevent late relapses. New ideas related to late relapses became apparent after considering the Dillekås et al., 2016 paper [10]. These authors analyzed data from Norway for breast cancer patients who had a mastectomy and delayed reconstruction of various durations. When they set time zero to the original mastectomy, they found a bimodal relapse pattern, as has been reported in many of our papers. Then, when they performed the same analysis for the same patients but with time zero set to date of reconstruction, they also found a bimodal relapse pattern. There are two important interpretations. First, it is any surgery rather than just cancer surgery that causes SI and exits of dormant cancer deposits with metastatic outcome. Second, and this only recently became obvious, if they did not analyze these data, the early relapses in the patients with time zero set to date of reconstruction would be considered ordinary late relapses—but we know they were triggered from dormancy to active growth by a surge of SI after cosmetic surgery. Judging from the data shown in Figure 1 from the Dillekås et al. paper, it is apparent that this is not a small effect. 

This needs to be confirmed, but it seems that if the patients undergoing delayed reconstruction had been given perioperative ketorolac, the relapse surge within three years of the reconstruction surgery would not have occurred. 

Might this information lead us to propose a way to reduce late relapses for breast cancer patients who are at risk of metastatic relapse? These patients would be a significant fraction (perhaps half) of current breast cancer survivors who number 3.8 million in the US. Extrapolating from this analysis of delayed reconstruction, it would seem that any breast cancer patient or survivor at risk of late relapse could benefit from using NSAID ketorolac when any planned surgery is conducted for a cosmetic or health issue. At this time, there are insufficient quantitative data to calculate the magnitude of this effect except to say it definitely is not small. These ideas should be testable with a clinical trial or perhaps reviews of breast cancer patient databases.

However, what can be done for breast cancer survivors who are injured by some unplanned chance event such as an automobile accident, fall, dental problem, headache, etc.? To estimate the magnitude of that problem, based on data from Wikipedia, there were 6 million car crashes in 2010 in the US which led to the estimated number of breast cancer survivors possibly injured by car accidents in that year to be 31 per day. To compare that unplanned event to a planned event, there were 48 million hospital surgeries performed in 2009 in the US.

We would need a panel of physicians and scientists who could monitor and advise changes to diet and drugs to reduce and keep SI at low base levels for patients at risk of late relapse. This will vary from patient to patient depending on many factors including dietary preferences and medical history. At least one member of this panel will be available at any time by phone or text to respond if a patient is planning a voluntary surgery or experiences an involuntary event that can precipitate a surge in SI. A relatively safe level of SI would probably be what can be achieved with a low inflammation diet [11]. A person with a relatively recent history of cancer and planning any surgery should probably use perioperative and post-operative ketorolac.

As an estimate of the time needed after an unplanned event to reduce SI to a safe low level, in non-small cell lung cancer, the time when the level of SI best predicts early relapse is the first day post operation [12]. Thus, we should plan on a few hours at most to make necessary adjustments after an SI stimulating event. Thus, our panel must have rapid access to the medical records of each person that we are monitoring. The subject would have to always carry appropriate anti-inflammatory drugs. After taking the appropriate drugs recommended by our panel member, the subject might have to go to a hospital Emergency Room or specialized center for monitoring and continued SI control for at least one week. The patient could also contact our panel for guidance. 

When is the end of the late-relapse risk period? For breast cancer, there have been reports of relapses over 30 years after initial treatment but that is very rare [13]. Perhaps 15–20 years is a reasonable end to the late-relapse risk period for breast cancer.

It is clear that timely data on SI would be highly important. As member of the Editorial Board of Biosensors, I have read many of the papers presented in that journal and consider these authors to be very inventive. Consider this communication not as a dare but a request. It would be an important development to have a convenient way of measuring SI without the need for a blood draw.

This has the potential to become a technology cooperation between a cancer research project based at Harvard and a biosensors or sensors team to help save lives for breast cancer patients. I have a provisional patent pending for this therapy. The patent pending will allow some entity to put a business together and have patent protection. The sensors or biosensors team will have the satisfaction of saving lives and generating some income. Before any work starts, *Sensors* and I need to negotiate terms of an agreement that covers what gets done and who gets what in return.

## Figures and Tables

**Figure 1:**
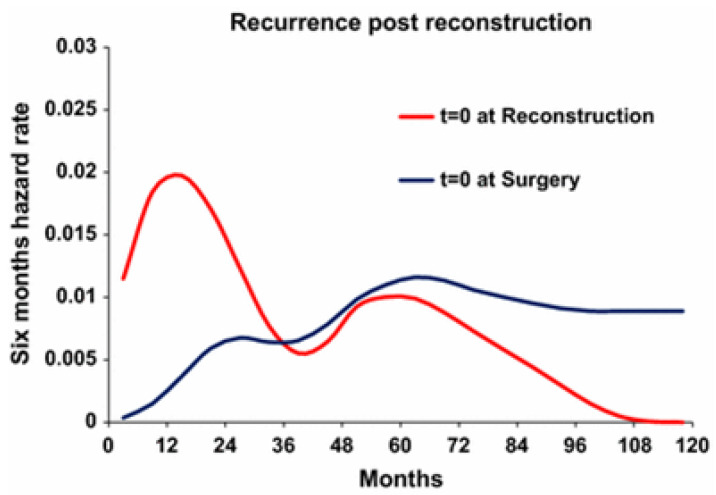
Recurrence pattern for the reconstructed patients (*n* = 312) with *T* = 0 set at reconstruction (*red line*) and at primary surgery (*blue line*). *X*-axis represents time in months. *Y*-axis represents six-month hazard rate. This figure is taken from Dillekås, Demicheli, Ardoino, Jensen, Biganzoli, and Straume. The recurrence pattern following delayed breast reconstruction after mastectomy for breast cancer suggests a systemic effect of surgery on occult dormant micrometastases [10].
